# The Romance of Cod-Liver Oil

**Published:** 1923-04

**Authors:** 


### THE ROMANCE OF COD-LIVER OIL.

Cod-liver oil is a nasty-looking fluid with an unpleasant smell and a vile taste. But lurking in its greasy yellow depths there is the very soul of romance, an unwritten record of murder and sudden death, and of an unbroken sequence of "life." Cod-liver oil has long been known as a valuable preventive of diseases such as rickets, but it is only of late years that these therapeutic properties have been traced to its remarkably high vitamine content. Weight for weight, cod-liver oil contains frcm 200 to 300 times as much vitamine as fresh butter. Vitamines can, apparently, not be created by the animal kingdom; generated in the vegetable kingdom, they can be passed through the animal kingdom by the simple process of one animal eating another. In a lecture recently given by Professor Poulsson to the Medical Society of Christiania, an interesting account was given of the history of the vitamines in cod-liver oil. They are generated in the tiny, unicellular algæ which grow in such profusion that the sea for many square miles assumes a muddy appearance. This vegetable plankton is consumed by plankton of the animal kingdom, and this animal plankton is in its turn consumed by small fish. Little fish have bigger fish upon their tracks to eat them, and cannibalism among fish proceeds until the small, arctic, salmon-like fish—*mollotus arcticus*—is reached.

### The Elusive Vitamine.

Full of much good vitamine, swarms of this fish seek refuge in the early winter months in the coastal waters west and north-west of Norway, pursued by millions of cod. During these months the *mollotus arcticus* is the chief source of food for the rapacious cod, whose vitamine requirements are, indeed, great. An average-sized cod lays from five to nine million eggs, each of which must have its share of vitamine. The cod having been caught, and oil having been extracted from its liver, it might have been thought that the vitamine had reached the last stage in its long and exciting journey. But, as Professor Poulsson has shown, the step from the cod to man need not necessarily be its last. He has found that if a breast-fed infant is developing unsatisfactorily, giving cod-liver oil to the mother may have a dramatically beneficial effect on the infant, which at once proceeds to grow and flourish. The Norwegian Government, realising what an important national asset cod-liver oil is, has instituted and financed a scheme of research with a view to adding to our knowledge of it, and it has already been proved at the Pharmacological Institute in Christiania that while well-bottled cod liver oil retains a high vitamine content even when three years old, it loses its vitamine if it is exposed to the air for a fortnight. Evidently vitamines are sensitive, elusive, not to say capricious things, and before we can harness them like electricity to our coach, we must learn much more about them than we know at present.

"The Lancet" concludes the hundredth year of its existence in October, and it is proposed to hold a dinner in celebration of the event. Dr. J. H. W. Laing and Mr. H. D. Gillies, 7 Portland Place, W.1, are honorary secretaries.

